# Evaluation of inhibitor activity of bacterial sialidase from *Clostridium perfringens* against Newcastle disease virus in the cell culture model using chicken embryo fibroblast

**DOI:** 10.5455/javar.2022.i600

**Published:** 2022-06-30

**Authors:** Ryan Septa Kurnia, Rahajeng Setiawaty, Ketut Karuni Nyanakumari Natih, Christian Marco Hadi Nugroho, Otto Sahat Martua Silaen, Silvia Tri Widyaningtyas, Simson Tarigan, Fera Ibrahim, Pratiwi Pudjilestari Sudarmono

**Affiliations:** 1 Doctoral Program in Biomedical Science, Faculty of Medicine, Universitas Indonesia, Jakarta, Indonesia; 2 National Veterinary Drug Assay Laboratory (NVDAL), Raya Pembangunan Gunung Sindur, Bogor, Indonesia; 3 Animal Health Diagnostic Unit, PT. Medika Satwa Laboratoris Kp. Kayumanis, Bogor, Indonesia; 4 Institute of Human Virology and Cancer Biology Universitas Indonesia, Jakarta, Indonesia; 5 Indonesian Research Centre for Veterinary Science, Bogor, Indonesia; 6 Department of Clinical Microbiology, Faculty of Medicine, Universitas Indonesia, Jakarta, Indonesia

**Keywords:** Cell culture, Clostridium perfringens, in vitro, inhibit, sialidase, virus

## Abstract

**Objective:**

The Newcastle disease virus (NDV) is an infectious disease that causes very high economic losses due to decreased livestock production and poultry deaths. The vaccine’s ineffectiveness due to mutation of the genetic structure of the virus impacts obstacles in controlling the disease, especially in some endemic areas. This study aimed to provide an alternative treatment for NDV infection by observing the viral replication inhibitor activity of *Clostridium perfringens* sialidase in primary chicken embryo fibroblast (CEF) cells.

**Materials and Methods:**

The virus was adapted in CEF monolayer cells, then collected thrice using the freeze–thaw method and stored at −20°C for the next step in the challenge procedure. *C.* perfringens crude sialidase was obtained, but it was further purified via stepwise elution in ion exchange using Q Sepharose^®^ Fast Flow and affinity chromatography with oxamic acid agarose. The purified sialidase was tested for its toxicity, ability to breakdown sialic acid, stopping viral replication, and how treated cells expressed their genes.

**Results:**

According to this study, purified *C. perfringens* sialidase at dosages of 187.5, 93.75, and 46.87 mU effectively hydrolyzes CEF cells’ sialic acid and significantly inhibits viral replication on the treated cells. However, sialidase dosages of 375 and 750 mU affected the viability of monolayer CEF cells. Interestingly, downregulation of toll-like receptor (TLR)3 and TLR7 (*p* < 0.05) in the sialidase-treated group indicates viral endocytosis failure.

**Conclusions:**

By stopping endocytosis and viral replication in host cells, sialidase from *C. perfringens* can be used as an alternative preventive treatment for NDV infection.

## Introduction

Newcastle disease virus (NDV), also known as avian paramyxovirus type 1, has been linked to significant economic losses due to lower livestock production and poultry mortality. The mortality and morbidity rates caused by viral infections can reach up to 100%, with symptoms of respiratory, nervous, and digestive disorders [1]. In Indonesia, NDV has been known since 1926 on the island of Java. Vaccination is the most effective disease prevention and control measure against viral infections, in addition to biosecurity management in livestock areas. Two types of vaccines are used to control ND in the field: inactivated and live attenuated vaccines [2]. However, based on the number of cases discovered throughout the year and the 70%–80% mortality rate, Indonesia remains an endemic location for ND [3]. 

The diversity of genotypes and the ease with which the virus mutates are one of the obstacles to controlling the disease [4,5]. The use of inactivated vaccines is known to be effective in preventing ND infection. On the contrary, generating an IgG antibody response takes at least 14 days to protect the host from viral infections in the field [6,7]. In addition, live attenuated vaccines in the field are more efficient because they can be applied by spraying or drinking water but cannot block field virus replication. This results in the shedding of the virulent NDV into the environment and leads to potential outbreaks among unprotected birds due to poor immune conditions or secondary infections [8,9]. Antiviral control mechanisms, such as rimantadine, amantadine, ribavirin, and oseltamivir, are costly and unsuitable for use in poultry. Furthermore, rapid mutation of genetic material affects the variance of subgenotypes in the virus’ structural components and viral protein and results in viral resistance to antiviral medications [10–12]. 

Research on the inhibition of viral infection by degrading receptors on host cells using enzymes derived from bacteria has been carried out previously. The enzyme was created by first using a recombinant fusion protein from the bacteria Actinomyces viscosus to prevent viral infections in the respiratory tract. This causes failure of the virus to attach to and enter host cells in vitro and *in vivo*, so that virus replication does not occur [13,14]. Worrall et al. [12] also proved that sialidase derived from *Clostridium perfringens* type A in a mixture of intranasal vaccines could protect poultry from avian influenza virus subtype H5N1 outbreaks. The addition of sialidase was carried out to degrade the sialic acid receptors of the host mucosal epithelial cells to prevent early viral infection through the respiratory tract [12]. Further research on the mechanism of replication inhibition by virus challenge on host cells has never been proven previously in vitro. Developing strategies to control viral infections is a challenge in biomedical science [15]. 

This study describes the potential of sialidase in inhibiting NDV replication in vitro using chicken embryonated fibroblast cells so that the mechanism of interaction of C. perfringens sialidase against viral infection and cell cytokine expression in vitro in the host cells can be revealed.

## Materials and Methods

### Ethical approval

This study made no use of live animals. The Ethics Committee of the Faculty of Medicine at the University of Indonesia (No. KET-1482/UN2.F1/ETIK/PPM.00.02/2020) had looked over and approved the methods used in this study.

### Identification, confirmation, and adaptation of NDV isolates

In this study, the virus strain chicken/1/78/MSL/19 used was an isolated archive from the NDV outbreak, which was stored in freeze-dried ampoules. The virus in freeze-dried ampoules was diluted with PBS and then inoculated onto the prepared monolayer cells in a 25 cm^2^ flask and incubated in a CO2 incubator at 37°C for 24–72 h. The infected monolayer cells in the flask were observed twice a day using an inverted microscope to see the cytopathogenic effect (CPE). Then, the virus was harvested by the freeze–thawing method thrice and centrifuged at 3000× *g* for 15 min. The supernatant containing the virus was then stored at −20°C for further genetic confirmation and 500 TCID50 calculation. Genetic confirmation of the virus originating from the supernatant was extracted using the Geneaid Viral Nucleic Acid Extraction Kit II and reverse transcription–polymerase chain reaction (RT-PCR) was carried out on the gene encoding the fusion NDV protein (Fus) with the primer sequences forward 5’-ATGGGCTCCAGACCTTCTACCA-3”and reverse 5’-CTGCCACTGCTAGT TGTGAT AATC-3” [16,17]. 

### Production, purification, and activity assay of sialidase

The medium for the cultivation of *C. perfringens *type A consisted of trypticase, yeast extract, cysteine hydrochloride, and NaCl 1.0%, pH 7.4. This bacterium was cultured under anaerobic conditions at 37°C overnight. During the production process, the pH condition is observed every 10 minutes to maintain the pH in the range of 7. The final culture was cooled and centrifuged to remove cells. The separated supernatant was then treated with a decrease to pH 5 to inactivate the toxin activity, which was then called crude sialidase. To concentrate the protein, ammonium sulfate precipitation was used, resulting in a brown product that was dialyzed against 20 mM Tris buffer, pH 8 [18]. The dialysate was further purified by stepwise elution in ion exchange utilizing Q Sepharose^®^ Fast Flow and affinity chromatography with oxamic acid agarose before being kept at −20°C [19]. Purified sialidase enzyme activity was observed using the Neuraminidase assay kit MAK121 (Sigma-Aldrich) with appropriate protocol procedures to obtain a quantitative value in U/ml.

### Preparation of chicken embryo fibroblast and primary culture cells

The use of chicken embryo fibroblast (CEF) is the “gold standard” for cultivating NDVs originating from poultry. These cells had come from chicken eggs with SPF embryos aged 9 days. The embryo from the carcass was separated mechanically and enzymatically to obtain a single fibroblast cell. The growth medium used contained 5% heat-inactivated fetal calf serum (FCS), 2% L-Glutamine, 2% sodium bicarbonate, and 1% antibiotic solution (penicillin, neomycin, and streptomycin). The cell suspension was then counted using a hemocytometer with a concentration of not less than 1×10^6^ cells/ml and inoculated onto a 96-well microplate. Incubation in a CO2 incubator at 37°C was carried out for 24 h and the growth of monolayer cells was observed using an inverted microscope [20]. 

### Cytotoxicity evaluation of sialidase

The toxicity of sialidase was tested by adding sialidase doses of 750 mU, 375 mU, 187.5 mU, 93.75 mU, and 46.87 mU to the CEF cell culture medium maintenance mixture, which contained 1% heat-inactivated FCS, 2% L-Glutamine, 2% sodium bicarbonate, and 1% antibiotic solution (penicillin, neomycin, and streptomycin). Sialidase was added to monolayer CEF cells in a 96-well plate and then incubated in a CO2 incubator at 37°C for 2 h. Sialidase was removed, replaced with a maintenance medium, and then reincubated in a CO2 incubator at 37°C for 48 h. Cell viability was observed with the CellQuanti-MTT Bioassay kit according to the manufacturer’s instructions. Absorbance data obtained from the MTT test were converted into the form of the percentage of living cells or cell viability, which can be calculated by the following formula: Viability cell percentage (%) = [(Absorbance of treatment cells − Absorbance background) / (Absorbance control cells − Absorbance background)] × 100% [21,22]. 

### Detection of sialic acid in cell surface

This method was carried out to detect the presence of sialic acid on the cell surface after administration of sialidase in several concentrations. Monolayer CEF cultured cells in 96-well microplates were treated with sialidase for 1 h at 37°C, then washed with PBS thrice. The cells were then blocked with the addition of 0.2% casein in PBS to stabilize the molecular bonds at the bottom of the microplate and reduce the background. The fixed cells were then washed with PBS 0.1% Tween 29 (PBST) and incubated for 1 h at 25°C with 20 gm/ml biotinylated *Maackia amurensis* lectin (GlycoMatrix™) to detect Neu5Ac (2,3)Gal sialic acid. Furthermore, washing with PBST was followed by secondary detection of lectin binding in cells using 5 gm streptavidin–HRP (Biolegend^®^) and incubation for 1 h at 25°C. Washing was carried out five times, followed by adding ABTS substrate (2,2’-Azinobis [3-ethylbenzothi-azoline-6-sulfonic acid]-diammonium salt). Absorbance readings were measured using a spectrophotometer at a wavelength of 405 nm, and the percentage of sialic acid was calculated using the following formula: 100% × [(Absorbance of treated cells − Background) / (Absorbance of cells without treatment − Background)]. Background control is well administered with streptavidin–HRP without adding lectins [23,24]. 

### Viral replication inhibition assay

The monolayer of CEF-grown cells on 96-well plates incubated at 37°C for 24 h produced 4 treatment groups. The groups were mock control, NDV inoculation control, sialidase-treated cells + NDV challenge, and sialidase combined antisialidase (oseltamivir) + NDV challenge. Cells were treated with sialidase by adding 100 µl of sialidase at various dilution dosages to the maintenance medium and incubated at 37°C for 2 h. It was then incubated for 24 and 48 h with 100 µl of 500 TCID_50_ NDV in maintenance medium. Using an inverted microscope, observations of the appearance of CPE in the NDV control group were made at 24-h intervals. Cells on the microplate were harvested, transferred to microtubes, and stored at −80°C for later RNA extraction [25,26]. 

### Real-time quantitative reverse transcription PCR

The harvested monolayer cells were washed, and then the cells were extracted using the ReliaPrep RNA cell miniprep system by Promega. The RNA concentration obtained was quantified using the QuantiFluor^®^ RNA System kit, Promega, according to the manufacturer’s procedure. Furthermore, the reading of the RNA concentration was carried out with the Quantus™ Fluorometer. The quantitative RT-PCR (qRT-PCR) method begins with converting RNA into cDNA using the ReverTra Ace cDNA synthesis kit (Toyobo). The relative qRT-PCR process was carried out with a total volume of 25 µl using the Kapa SYBR^®^ Fast Master Mix Kit (Roche) using specific primers for toll-like receptors (TLRs) and interferons (IFNs) (Table 1). As for the housekeeping gene, glyceraldehyde-3-phosphate dehydrogenase was used to normalize the expression of the target gene [27]. At the end of the amplification process, a melt curve analysis was carried out to confirm the specificity of the green SYBR PCR signal. The relative expression level was observed based on the cycle threshold (CT) value of the target gene normalized with the housekeeping gene. The expression results are interpreted based on an increase or decrease in fold change in gene expression compared to controls [25].

Estimation of viral copy number was carried out using absolute qRT-PCR with forward and reverse primers for gene F from NDV in the forward sequence 5’-TTGATGGCAGGCCTCTTGC-3” and the reverse sequence 5’-GGAGGATGTTGGCAGCATT-3”. Dilutions in multiples of 10 against positive control NDV RNA ranging from 1 to 1 × 10^6^ copies/reaction were used to obtain a standard curve [28]. The CT value of the amplification results was converted to a viral copy number based on calculating the slope and intercept values of the standard curve.

**Table 1.** Primer sequences for qRT-PCR gene expression of CEF primary cells [22].

Gene	Primer sequence	GenBank accession number
**GAPDH**	(F) 5’-CCTCTCTGGCAAAGTCCAAG -3”	NM_204305
	(R) 5’-CATCTGCCCATTTGATGTTG -3”	
**TLR3**	(F) 5’-ACAATGGCAGATTGTAGTCACCT-3”	NM_001011691
	(R) 5’-GCACAATCCTGGTTTCAGTTTAG-3”	
**TLR7**	(F) 5’-TGTGATGTGGAAGCCTTTGA-3”	DQ780342
	(R) 5’-ATTATCTTTGGGCCCCAGTC-3”	
**IFN-α**	(F) 5’-ATGCCACCTTCTCTCACGAC-3”	EU367971
	(R) 5’-AGGCGCTGTAATCGTTGTCT-3”	
**IFN-β**	(F) 5’- CCTCAACCAGATCCAGCATT-3”	AY831397
	(R) 5’- GGATGAGGCTGTGAGAGGAG-3”	
**IFN-γ**	(F) 5’- TGAGCCAGATTGTTTCGATG-3”	DQ906156
	(R) 5’- CTTGGCCAGGTCCATGATA-3”	

## RESULTS

### Synthesis, purification, and potential activity of C. perfringens sialidase

**Figure 1.**
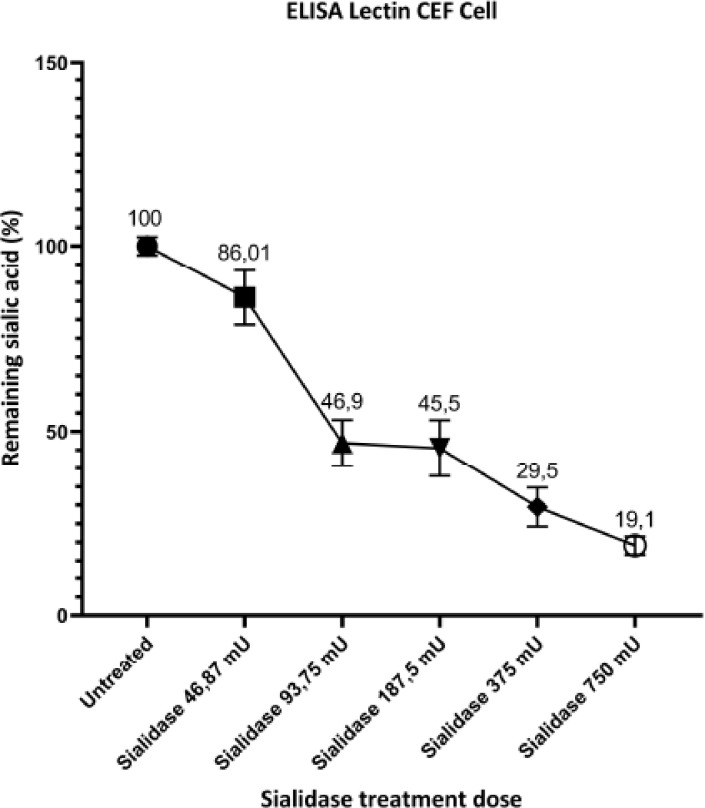
Sialic acid removal from the surface of CEF primary cells. The remaining sialic acid was detected by enzyme-linked lectin assay using biotinylated lectins after treatment with various doses of sialidase for 2 h at 37°C incubation.

Sialidase was synthesized from *C. perfringens* type A with NanI encoding the sialidase gene. The purified sialidase showed that the protein fraction appeared to have a molecular weight of 56 kDa with a specific activity of 75 U/mg. This fraction was stable at pH 5 and 7 for 72 h at 37°C with a gradual decrease in activity to meet the requirements for *in vitro* testing on primary CEF cells. Based on the presence of sialic acid on the cell surface after sialidase administration, it was able to hydrolyze sialic acid receptors from the primary cell surface of CEF cells until 19.1% of the sialic acid remained due to the administration of 750 mU of sialidase (Fig. 1). The amount of sialic acid remaining on the surface of the cells increases with a decrease in the dose of sialidase administration to primary CEF cells. The amount of sialic acid remaining increased with a reduction in the quantity of sialidase administration to primary CEF cells; for example, 375 mU of sialidase 29.5% sialic acid and 187.5 mU of sialidase 45.4% sialic acid remained on the surface of the cells.

### Cell viability assay of CEF cells posttreatment by sialidase

**Figure 2.**
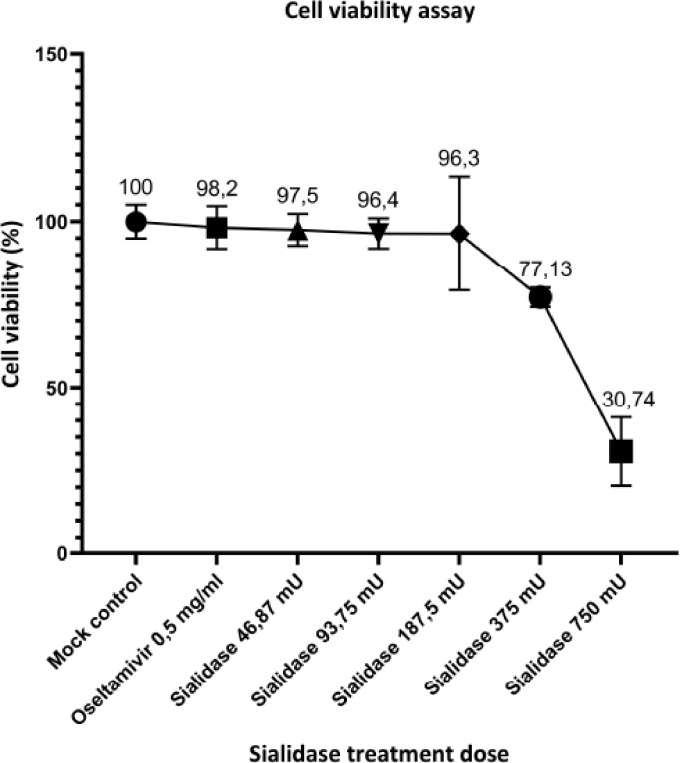
Cell viability assay of sialidase and oseltamivir posttreatment. Cell viability was measured to determine the toxicity of sialidase and oseltamivir MTT assay. In an initial approach, CEF cells were treated with various doses of sialidase and oseltamivir for 48 h.

MTT assays performed cytotoxicity evaluations using viability assays in CEF cell culture of this substance after cell treatment with various doses of sialidase and 0.5 mg/ml oseltamivir. Sialidase at the highest dose of 750 mU caused a decrease in cell viability for the remaining 30.74%. In comparison, at a lower dose (375 mU), the percentage of remaining cell viability was 77.13% compared to the mock control cell group. Sialidase exhibited decreased cell viability, with a dose of 750 and 375 mU μg/ml. However, no significant toxicity was found in a treated cell with a dose of 187.5–46.87 mU and 0.5 mg/ml oseltamivir by the percentage of cell viability above 95% (Fig. 2). Sialidase showed a concentration–response relationship since cell viability decreased gradually with the increase in its concentration, as shown in Figure 2.

### Inhibitory effects of sialidase on virus replication in primary cells

**Figure 3.**
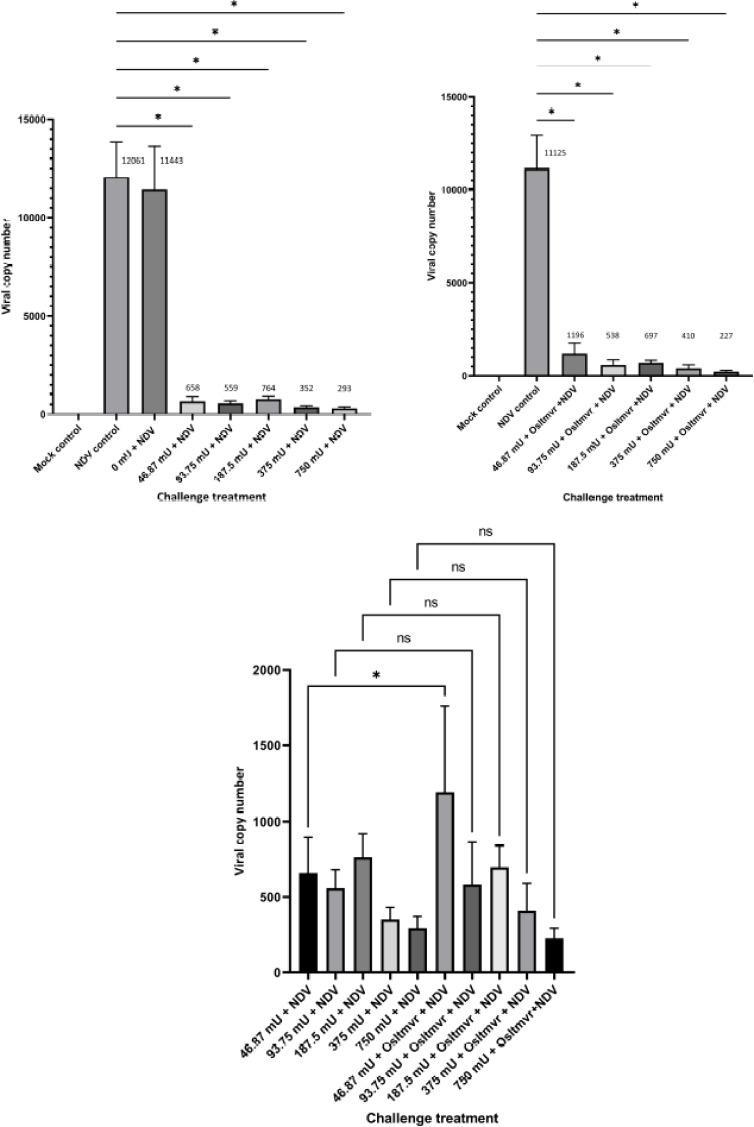
Viral replication inhibition on CEF cells. (A) Inhibitory effect of NDV replication determined by viral copy number of sialidase-treated cells. (B) Viral copy number of sialidase + oseltamivir-treated cells. (C) Comparison of viral copy numbers between the competitive inhibition sialidase treatment and sialidase + oseltamivir

Observation of the viral copy number in cells postinfection showed an increase in the control of NDV. An increase did not follow the rise in the number of viruses in the NDV control group and the number of viruses in the sialidase treatment group. The amount of viruses was observed based on the viral copy number in CEF cells, which showed that the administration of sialidase at a dose of 750–46.87 mU was able to significantly inhibit NDV replication (p < 0.05) compared to the control group of NDV (Fig. 3A). Treatment with inactive sialidase (0 mU) was carried out to prove that there was no intervention from other enzymes that inhibited NDV replication. In addition, anti-sialidase (oseltamivir) at several doses of sialidase showed no significant impact on the ability of sialidase to inhibit viral replication compared to the control group of the NDV. Thus, an observation was made on the comparison of viral copy numbers between the competitive inhibition sialidase treatment and sialidase + oseltamivir. The results showed that there was a significant difference (p < 0.05) at the lowest dose of 46.87 mU compared to the group without the addition of oseltamivir (Fig. 3B). The difference was in the form of an increase in viral copy number, which indicated a disturbance in the ability of sialidase by oseltamivir in a dose-dependent manner to inhibit viral replication in CEF cells (Fig. 3C).

**Figure 4.**
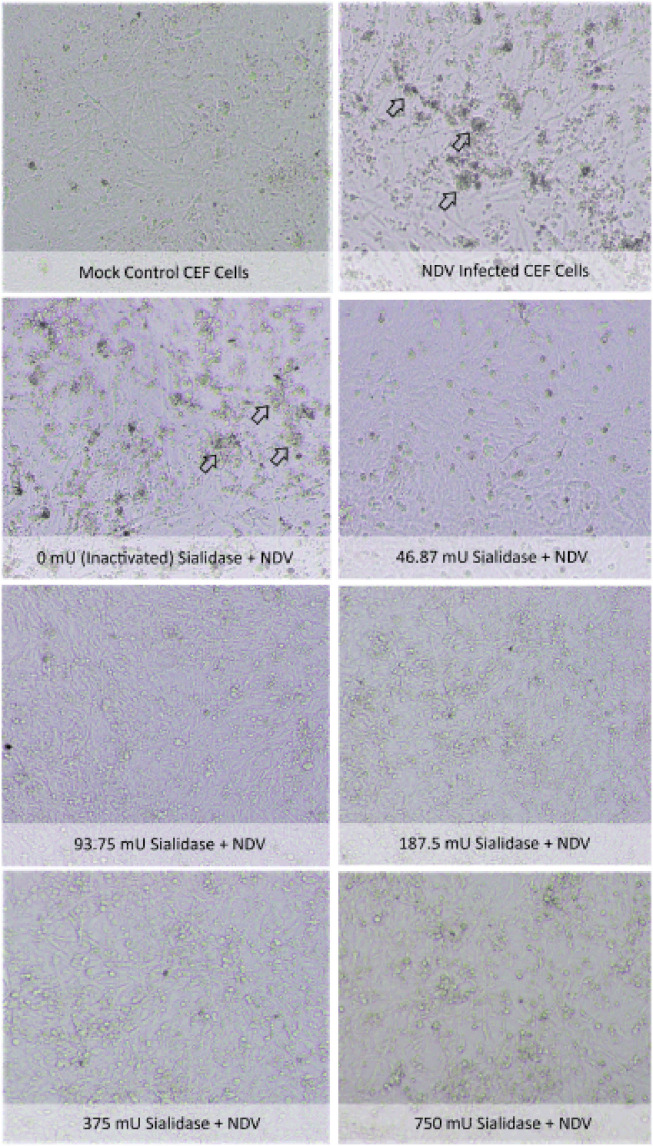
Microscopic observations of treated CEF cells. Observations on the structure of fibroblasts were observed using an inverted microscope with 400× magnification. The appearance of CPE in the form of multinucleated *giant cell *on NDV-infected CEF cells indicates the growth of the NDV. With the administration of 750 mU, disruption of cell–cell and cell–matrix adhesions was observed so it causes fibroblast cells appearing less frequently than mock control CEF cells.

Microscopic observations showed the difference between normal CEF cells without treatment and CEF cells treated with sialidase. At a dose of 750 mU sialidase, cell damage was observed in the structure of intercellular fibroblasts due to disruption of cell–cell and cell–matrix adhesions. It results in fibroblast cells appearing less frequently than mock control CEF cells due to the formation of cell gaps and loss of large areas of the monolayer. Meanwhile, CPE generated by NDV virus replication was associated with alterations in fibroblast organization in the form of multinucleated giant cells in CEF cells infected with NDV that were not treated with sialidase (Fig. 4).

### Expression of IFNs and TLRs on primary CEF cells

After the competitive inhibition treatment, the expression of IFNs and TLRs on CEF cells was used as a parameter for interactions that occur in the cells. The expression levels of TLR3 and TLR7 were observed in CEF NDV-challenged cells treated with various doses of sialidase 48 h after infection. TLR3 and TLR7 expressions were 8.4 and 1.9-fold higher in the control group of cells infected with NDV, respectively, compared to normal CEF cells. TLR expression in sialidase-treated cells at various doses showed significant differences (p < 0.05) against TLR3 and TLR7 compared to the group of cells infected with the NDV without treatment. However, based on observations on the graph, it shows a pattern of upregulation of TLR3 expressions, especially in treatment with the upregulated sialidase doses of 93.75 and 46.87 mU by 6.22 and 6.17-fold, respectively. Meanwhile, TLR7 observations showed a significantly downregulated expression (p < 0.05) at almost all doses of sialidase. Expressions of TLR3 and TLR7 mediate an activated antiviral immune response due to the entry of viral RNA into cells.

**Figure 5.**
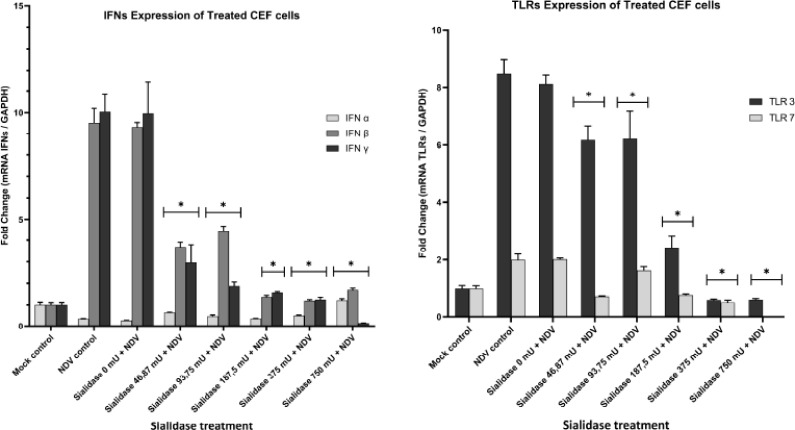
Observation of IFNs gene expression of competitive inhibition challenge. (A) Gene expressions of IFN-α, IFN-β, and IFN-γ in CEF cells that were given several doses of sialidase and then challenged with NDV. (B) TLR3 and TLR7 gene expression in CEF cells given several doses of sialidase then challenged with ND virus.

Observations on IFNs showed an upregulated expression of IFN-β and IFN-γ by 9.5- and 10-fold in the control group of cells infected with NDV compared to normal cells. Cytokine expression in cells treated with sialidase at all doses showed no significant upregulation (*p* < 0.05) in IFN-β and IFN-γ compared to the group of cells infected with NDV without sialidase treatment. Meanwhile, based on the observation of the IFN-α gene, the control group of cells infected with the NDV shows a downregulated expression of 0.3-fold compared to normal control cells (Fig. 5A and B). However, cells treated with sialidase showed significant differences in IFN-α expression with the group of cells infected with the NDV. This indicates that sialidase can interfere with regulating the expression of IFNs and TLRs cytokines, thereby inhibiting the replication of NDV in CEF cells.

## Discussion

In recent work, *in vitro* models of CEF cell culture have demonstrated the ability of *C. perfringens* bacterial sialidase activity to hydrolyze sialic acid receptors and significantly inhibit NDV replication by interfering with the regulation of TLRs and IFNs expression in treated cells. These findings reveal that *C. perfringens* sialidase can inhibit NDV replication through a complex mechanism involving an immune response that has not been demonstrated in previous studies. This study supports earlier studies regarding the ability of sialidase in intranasal vaccine mixtures to prevent H5N1 avian influenza infection in poultry [12]. This study also confirms that *C. perfringens* bacterial sialidase competes with viral sialidase by hydrolyzing CEF cell sialic acids. The presence of sialic acid on the primary CEF cell surface decreased after sialidase administration. Administration of sialidase at the highest dose caused a decrease in sialic acid so that 19.1% remained on the cell surface. In comparison, the administration of sialidase at a lower dose increased the amount of sialic acid by 29.5%. This indicates that the dose of sialidase impacts the amount of hydrolyzed sialic acid. Sialidase is an enzyme that hydrolyzes the terminal-linked sialic acid from various glycoproteins, glycolipids, and oligosaccharides, which is the first step in glycoconjugate degradation [29,30]. This enzyme is found in viruses, bacteria, and parasites that mostly act as virulence factors. Inhibiting virus sialidase in viral infection disease is thought to be a potential antiviral agent [31].

Previous studies have indicated that sialidases are significant virulence factors that promote *C. perfringens* pathogenesis by modifying the surface of MDCK cells, resulting in increased ETX binding and cytotoxicity [32]. Yet, no pathogenic role for these enzymes that remove terminal sialic acid residues from glycoproteins and glycolipids has ever been found [33]. In this study, we reveal that sialidase at the maximum dose-reduced cell viability demonstrates a concentration–response connection, as cell viability decreased progressively as concentration increased. These findings are consistent with a previous report, which found that treating monolayer cells with a high dose of *C. perfringens* sialidases causes cells to lose cell–cell connections and disperse individual cells. This shows that removing sialic acid carbs from the cell surface could cause slight changes in glycosylation patterns, leading to significant changes in how the cell works [34].

TLR gene expression is one of the innate immunity systems that use germline-encoded pattern recognition receptors (PRRs) for the early identification of microorganisms. PRRs recognize microbe-specific molecular signatures known as pathogen-associated molecular patterns (PAMPs). PRRs activate downstream signaling pathways that produce inflammatory cytokines, IFNs, and other mediators, inducing innate immune responses [35,36]. The upregulation of TLR expression in NDV-infected CEF cells in the current study follows a previous study which found the expression level of TLR3 and TLR7 significantly elevated in NDV-infected cells [25]. In response to viral infection, viral ssRNA is recognized by TLR7. On the contrary, TLR3 recognizes viral dsRNA *or* genomic structures or is generated during viral RNA replication intermediates in virus-infected cells [37–39]. Interferon (IFN) type I and type II expressions through recognition of PAMPs by putative pattern recognition receptors (PRRs), such as TLRs, are essential for regulating the antiviral immune response in host cells [40,41]. In this study, the expression of IFN signaling components at 48 h revealed that the upregulation in IFN-β and γ occurred in cells infected with the NDV. On the contrary, in IFN-α, there was a downregulation. Previous studies have shown that large amounts of IFN are produced by various host cells when infected with NDV [42,43]. 

The administration of sialidase treatment on NDV-infected cells seemed to interfere with the expression of TLR7 and TLR3, resulting in a suppression of expression compared to control cells with NDV infection. However, there appears to be an increase in the expression pattern of TLR3 in the low-dose sialidase treatment group, indicating incomplete hydrolysis of sialic acid so that the virus enters through the remaining sialic acid into cells. Furthermore, the mechanism of putative pattern recognition receptors based on IFN expression causes lower expression of IFN-β and γ in the group treated with sialidase. Likewise, based on observations of viral replication, there was a drastic decrease in the viral copy number of gene F in the sialidase treatment group. The number of copies of the fusion gene (F) indicates the amount of NDV replication in CEF cells. On the other hand, the F protein is a fundamental aspect that plays an essential role in viral virulence and tissue tropism [44]. The NDV genes are organized in the following order based on genomic RNA: 3’-NP-P-M-F-HN-L-5”. These sections are *cis*-acting regulatory elements involved in genomic and antigenomic RNA replication, transcription, and packaging. The beginning and end of each gene are conserved transcriptional regulatory sequences, known as the “gene start” and “gene end”, respectively [45,46]. Recent work is in line with previous studies that proved the presence of sialic acid on the cell surface can increase the efficiency of viral infection. On the contrary, sialic acid on the cell surface is removed by sialidase; it reduces viral binding and replication to host cells. However, incomplete hydrolysis of sialic acid may lead to viral replication through the sialic acid residues on the cell surface [23,47]. The expressions of TLRs and IFNs observed in this study describe signaling molecules and cytokine interactions that occur in cells and are directly related to viral infection mechanisms. Future studies on additional investigations using the animal challenge viral model will provide further insight to offer a comprehensive understanding of the innate immune response to evaluate the efficacy of *C. perfringens* sialidase.

## Conclusion

The study has revealed that sialidase derived from *C. perfringens* inhibits NDV replication and does not exhibit significant toxicity at effective concentrations *in vitro*. Sialidase effectively hydrolyses sialic acid receptors on the cell surface to reduce viral binding and prevent viral endocytosis. Endocytosis of NDV through the remaining sialic acid receptors can still cause an increase in the expression of TLRs. However, this expression will induce the expression of IFNs, causing interference with viral replication. Therefore, the sialidase represents a promising prophylaxis treatment for the poultry industry that may prevent NDV infection.

## List of abbreviations

CEF, chicken embryo fibroblast; FCS, fetal calf serum; h, hour; IFN, interferon; NDV, Newcastle disease virus; TLR, toll-like receptors; MTT assay, [3-(4,5-dimethylthiazol-2-yl)-2,5-diphenyltetrazolium bromide] assay;PBS, Phosphate Buffer Saline
